# Lack of HCAR1, the lactate GPCR, signaling promotes autistic-like behavior

**DOI:** 10.1186/s12964-023-01188-z

**Published:** 2023-11-09

**Authors:** Mohammad Ali Mohammad Nezhady, Gael Cagnone, Jean-Sébastien Joyal, Sylvain Chemtob

**Affiliations:** 1 Program in Molecular Biology, Faculty of Medicine, University of Montreal, Montreal, QC H3C 3J7 Canada; 2 Department of Pediatrics, Sainte-Justine University Hospital Research Center, University of Montreal, Montreal, QC H3T 1C5 Canada

**Keywords:** Lactate, HCAR1, GPCR, Signaling, Autism spectrum disorder, Autistic-like behavior, Anxiety

## Abstract

The GPCR HCAR1 is known to be the sole receptor for lactate, which modulates its metabolic effects. Despite its significant role in many processes, mice deficient in HCAR1 exhibit no visible phenotype and are healthy and fertile. We performed transcriptomic analysis on HCAR1 deficient cells, in combination with lactate, to explore pathophysiologically altered processes. Processes such as immune regulation, various cancers, and neurodegenerative diseases were significantly enriched for HCAR1 transcriptomic signature. However, the most affected process of all was autism spectrum disorder. We performed behavioral tests on HCAR1 KO mice and observed that these mice manifest autistic-like behavior. Our data opens new avenues for research on HCAR1 and lactate effect at a pathological level.

Video Abstract

**Supplementary Information:**

The online version contains supplementary material available at 10.1186/s12964-023-01188-z.

## Main text

Lactate has been extensively studied for its various effects, including cell migration, immune modulation, angiogenesis, cytoprotection, and many others. Its mechanism of action remained unknown until the discovery of its receptor HCAR1 [1]. Initially identified as GPR81, HCAR1 was discovered in 2001 [2] but remained an orphan GPCR until 2009, when lactate was found to be its endogenous ligand [3]. Studies on HCAR1-deficient mice demonstrated that lactate inhibits lipolysis in an insulin-dependent, para-autocrine manner through HCAR1 activation [4]. Although the highest expression level of HCAR1 is detected in adipocytes, it is expressed in almost every tissue tested [3]. Accordingly, a variety of physiologic roles of HCAR1 have been observed.

Lactate produced during labor in the uterine tissue reduces inflammation via HCAR1 activation [5]; lactate also reduces inflammation by modulating Toll-Like receptor signaling through HCAR1 [6]. HCAR1 activation by lactate produced in intestinal microbiota promotes intestinal stem cell proliferation and epithelial development by activation of Wnt/β-catenin pathway [7]. HCAR1 activation using different agonists leads to hypertension by regulating the endothelin vasopressor system in the kidneys [8]. In brain, HCAR1 signaling downregulates neural basal activity and firing frequency [9], while separately enhances angiogenesis by inducing VEGF [10]. Notably, HCAR1 is predominantly expressed in neurons of the cerebral cortex and hippocampus, and participates in postnatal microvascular development [11]. Whereas in astrocytes, HCAR1 promotes the expression of neurotrophic factors [12] and neurogenesis in the ventricular-subventricular zone in the brain [13].

**Fig. 1:**
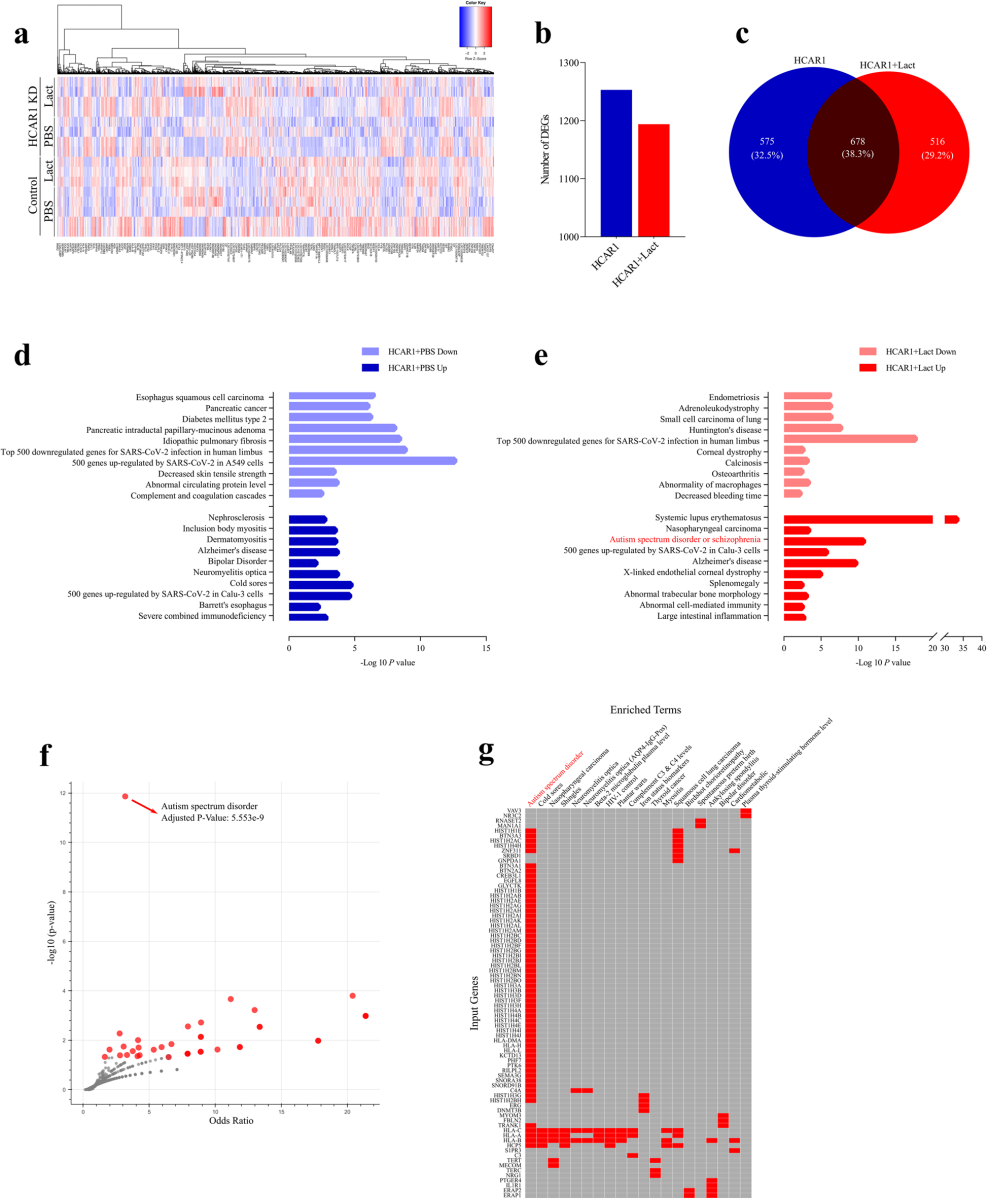
HCAR1 transcriptomic signature modulates wide range of pathologies. **a** Heatmap of HCAR1 RNA-seq in HeLa cell lines. KD and scrambled shRNA cells were treated with PBS or lactate and subjected to RNA-sequencing. **b** Number of DEGs through HCAR1 with and without lactate treatment. **c** Venn diagram showing the overlap of DEGs through HCAR1 with or without lactate. **d** Disease association with DEGs in HCAR1 PBS treated groups. HCAR1 + PBS down: downregulated genes upon HCAR1 KD in PBS treated cells. HCAR1 + PBS up: upregulated genes upon HCAR1 KD in PBS treated cells. **e** Disease association with DEGs in HCAR1 lactate treated groups. HCAR1 + Lact down: downregulated genes upon HCAR1 KD in PBS treated cells. HCAR1 + Lact up: upregulated genes upon HCAR1 KD in PBS treated cells. **f** Volcano plot for DEGs specific to HCAR1 + lact group (the 516 genes in c). **g** Clustergram showing DEGs associated with each term. DEG: differentially-expressed genes; KD: knockdown

Despite these wide-ranging roles of HCAR1, mice lacking this receptor are devoid of visible phenotypic changes, and are healthy and fertile. In order to explore specific phenotypic features of HCAR1 deficiency, we performed whole transcriptomic analysis of the HCAR1 signaling signature. We treated HCAR1-expressing and knocked-down HeLa cells (as a model cell line) with lactate (10 mM for 6 h) or PBS, and performed RNA-sequencing to identify potential pathophysiologic alterations in HCAR1-deficient animals (Fig. 1a-c). While more than 1200 genes were differentially regulated in HCAR1 knocked-down cells, a smaller number of genes were regulated by the receptor stimulated with lactate (Fig. 1b). Interestingly, approximately half of the genes in both lactate stimulated and unstimulated states were shared, while the other half of the genes were unique to either PBS or lactate treatments (Fig. 1c). This infers that HCAR1-regulated genes are not solely modulated through lactate signaling, but that basal activity of HCAR1 may also exert some form of gene regulation.

Using gene ontological analysis, we focused on pathological/physiological processes that could be affected by HCAR1 signaling, rather than cellular or molecular processes regulated by this receptor. Various diseases were associated with transcriptomic signature of HCAR1, whether stimulated or not with lactate. Under basal HCAR1 transcriptomic activity (PBS treatment), we observed associations with different cancers, type 2 diabetes, certain immunological disorders and the blood coagulation pathway (Fig. 1d). In addition, HCAR1-dependent differentially-regulated genes were significantly shared with the top 500 genes that are altered in SARS-CoV-2 infection (Fig. 1d). Notably, the lactate-treated group also showed a strong correlation with gene expression changes observed in SARS-CoV-2 infection (Fig. 1e). Interestingly, a variety of neurologic disorders such as Alzheimer’s disease, bipolar disorder and neuromyelitis optica stood out (Fig. 1d). Upon lactate stimulation, additional neurologic disorders were also linked to the HCAR1-mediated transcriptional network, such as Huntington’s disease and adrenoleukodystrophy (ALD) (Fig. 1e). However, among neurologic ailments, Autism Spectrum Disorder (ASD) was the most significantly process affected by lactate signaling through HCAR1 (Fig. 1e,f); lactate was found to down regulate many of the genes involved in the autism syndrome (Fig. 1g).

**Fig. 2:**
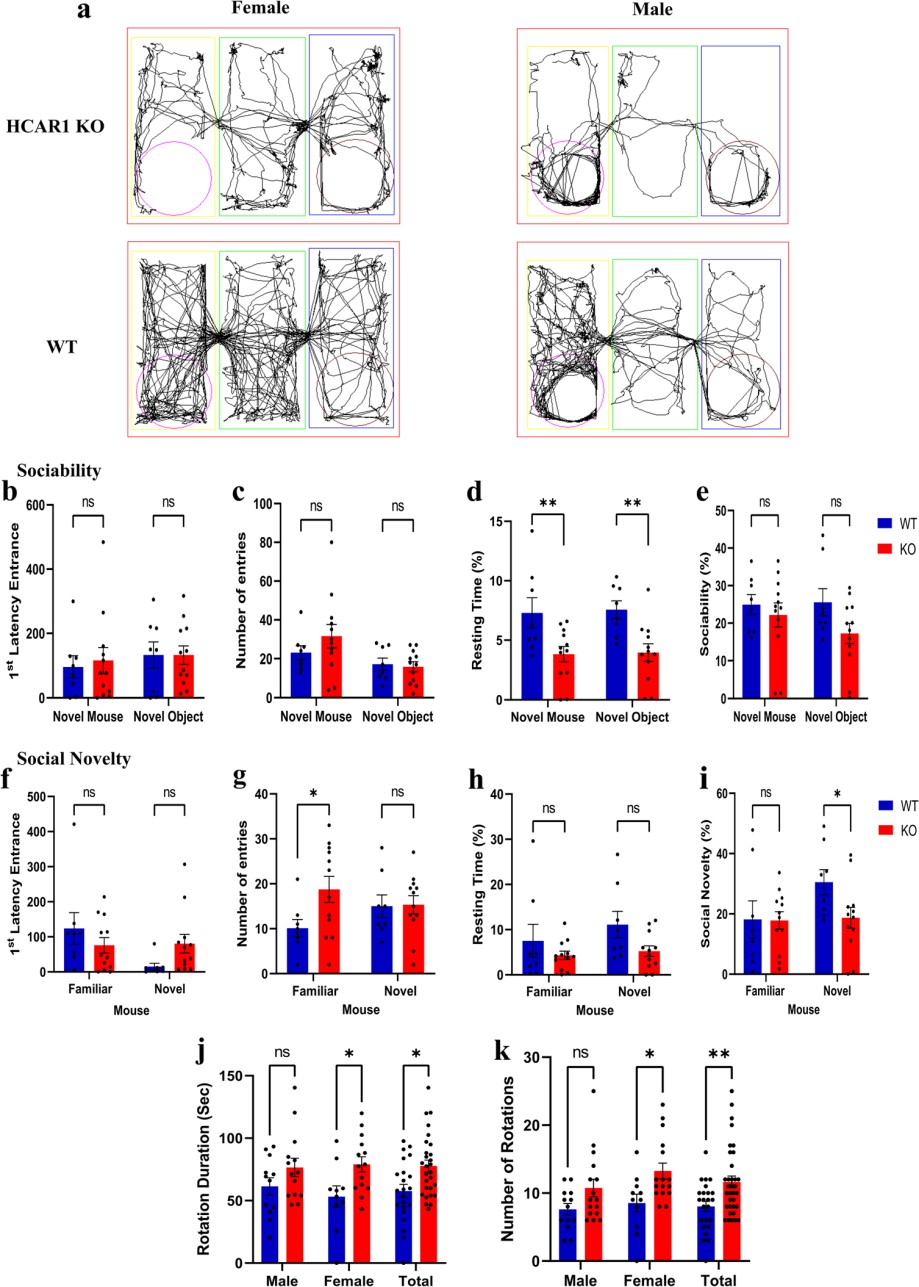
HCAR1-deficient mice exhibit altered social behavior. 3-chamber social test for social behavior: **a** Representative images showing subject mouse movement from social novelty phase of the test (3^rd^ phase). **b-e** Quantitative behavioral measure for sociability phase of the test (2^nd^ phase); 1^st^ latency to enter (**b**) and the number of entries (**c**) in the interaction zones and the sociability did not show any changes between HCAR1 KO and WT mice. However, KO mice spent less time in the chambers with novel mice or the chamber with novel object (**d**) indicating lower curiosity level in KO animals. **f-i** Quantitative behavioral measure for social novelty phase of the test (3^rd^ phase); 1^st^ latency to enter (**f**) and the resting time (**h**) in the interaction zones in the social novelty phase of the test did not show any changes between HCAR1 KO and WT mice. However, KO mice spent significantly more time in the interaction zone with the familiar mice (**g**) and had an overall lower social novelty score (**i**). **j-k** Total time that mice spent rotating clock or counter clockwise (**j**) and total number of clock or counter clockwise rotations (**k**) in the whole 3 phases of the test indicate that KO mice exhibit a higher rate of repetitive behavior. Data are mean ± s.d. from *n* = 12 for KO animal and *n* = 8 for WT animal. Analysis of Variance (ANOVA) was followed by Bonferroni post hoc correction test with * *P* < 0.05, ** *P* < 0.01 significance levels

We therefore proceeded to explore if HCAR1-deficient mice exhibited features of ASD given the presence of endogenous lactate in the brain. We performed 3-chamber social test on HCAR1 KO and WT mice. The 3-chamber social test measures animal sociability and social novelty seeking behaviors, which are among the main characteristic behavioral deficits in ASD [14, 15]. HCAR1-deficient mice spent significantly less time with either of the object (empty cage) or the novel mouse in the sociability phase, indicative of lower interest in exploring novel stimuli (i.e., curiosity) (Fig. 2d). While the HCAR1-KO mice exhibited altered behavior in this phase, other criteria for sociability were not significantly affected by the deficiency of HCAR1 (Fig. 2b,c,e). However, during the social novelty phase, HCAR1-KO mice visited the familiar mouse more often than the new mouse, and generally scored lower for social novelty behavior compared to WT mice (Fig. 2g,i). In addition, HCAR1-KO mice spent more time spinning on the spot—an indicator of a repetitive behavior as seen in ASD (Fig. 2j,k).

**Fig. 3:**
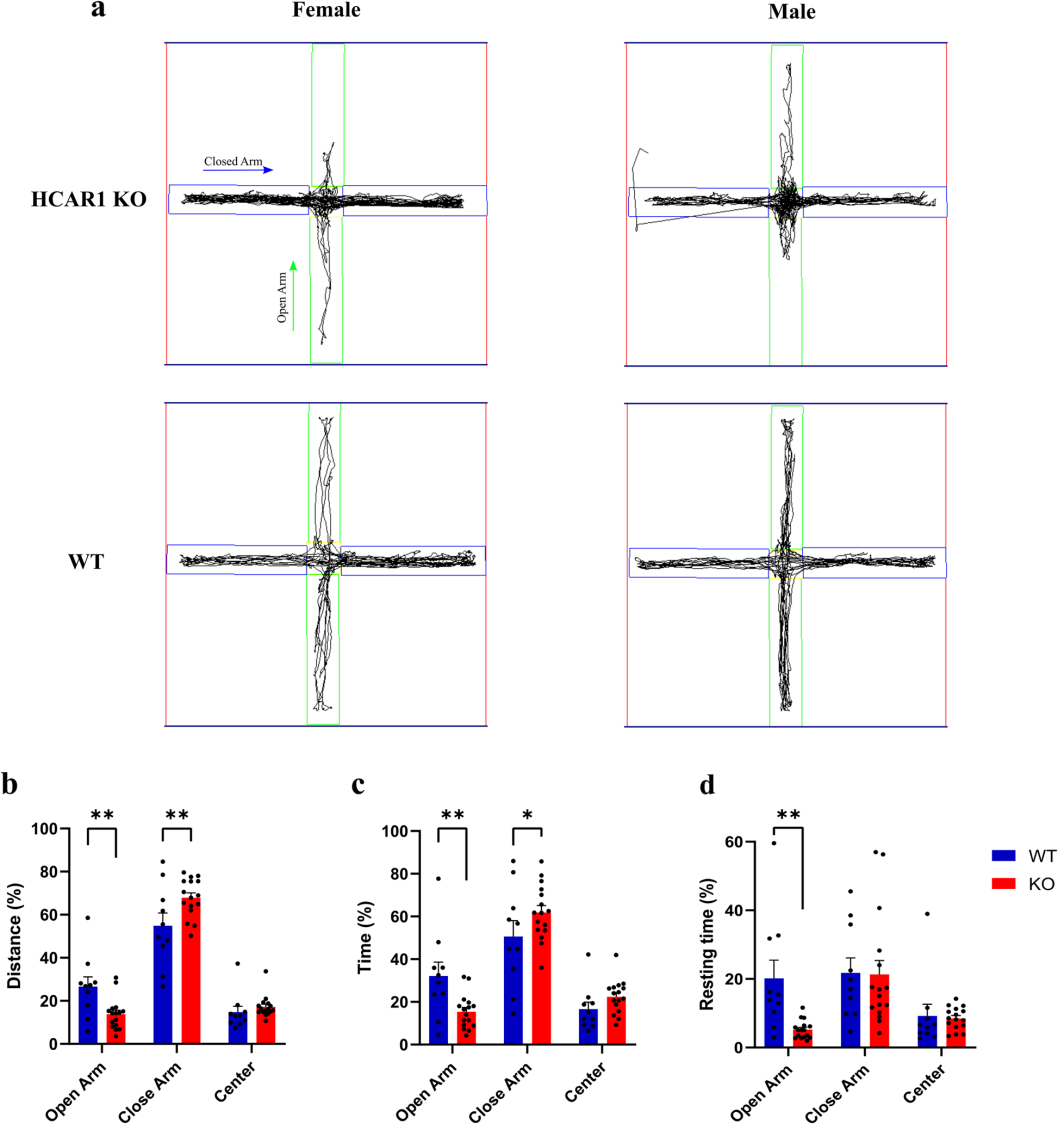
HCAR1-deficient mice exhibit anxiety-like behavior. Elevated plus maze test for anxiety-like behavior: **a** Representative images showing subject mouse movement in open and closed arms of the maze. **b-d** Quantitative behavioral measures for anxiety-like behaviors. HCAR1 KO mice traveled less distance (**b**), spent less time (**c**) and rested less time (**d**) in the open arm of the maze compared to close arm, indicating KO mice have more anxiety than WT. Data are mean ± s.d. from *n* = 16 for KO animal and *n* = 10 for WT animal. Analysis of Variance (ANOVA) was followed by Bonferroni post hoc correction test with * *P* < 0.05, ** *P* < 0.01 significance levels

We next evaluated the anxiety level of HCAR1 deficient animals, as a strongly associated co-morbid symptom of ASD, by performing elevated plus maze test [16]. HCAR1 KO animals exhibited aversion to the open arms of the maze (Fig. 3a). Accordingly, they traveled shorter distances in the open arms of the maze while preferring to move along the closed arms (Fig. 3b,c,d). The KO mice spent shorter times in the open arms compared to WT animals and mostly stayed longer in the closed arms (Fig. 3c). Even the resting time was significantly shorter in the open arms for KO mice (Fig. 3d). Overall, these features of HCAR1-deficient animals display anxiety-like behavior, pointing to a significant role for HCAR1 in regulating genes implicated in behavior which otherwise leads to autistic-like behaviors. Of relevance, HCAR1 KO mice presented no locomotor deficiencies, further attesting to altered behavioral patterns (Fig Supp. 1 and 2). There were also no sex differences for sociability, social novelty, and anxiety tests. Altogether, HCAR1-deficient animals exhibit reduced social behavior with increased repetitive and anxiety-like behaviors, pointing to a significant role for HCAR1 signaling in neural modulation of activities involved in regulating autistic spectrum phenotypes.

The role of HCAR1 in neurons and brain tissue has been explored [9–11]. The metabolism of astrocytes is to a large extent based on glycolysis resulting in accumulation of lactate (as end product), which seems to enhance their plasticity [12]. In neurons, HCAR1 reduces cell excitability [17]; conversely, neurons of HCAR1-deficient mice display higher basal activity [18]. Our data reveal that the absence of HCAR1 signaling axis along with the augmented brain activity could promote autistic-like behavior. Increased neural excitability in brain regions controlling sensory, social and emotional behavior have been linked to autism [19]. It would be tempting to speculate that lack or disruption of HCAR1 signaling could participate in hyperactive neural firings that contribute to manifestations of autism. Depending on the brain region, this disruptive signaling could be displayed through different features and degree of autistic-like behaviors. Consistent with this notion, although our data fails to reveal changes in some parameters of sociability features in mice lacking HCAR1, brain regions controlling social novelty behavior could exhibit altered function due to silencing of HCAR1 signaling pathway. Accordingly, modulation of HCAR1 could potentially serve as a therapeutic target for autism, opening new avenues for investigation in this context.

As expected, given the involvement of HCAR1 in both neural and immune cell functions, many diseases associated with the HCAR1-regulated transcriptome were neurological and immunological disorders (Fig. 1d, e). An interesting observation was the similarity of HCAR1-deficient transcriptomic signature with the altered transcriptome of SARS-CoV-2 infection, which could at least partially arise from immune related functions of HCAR1, and the metabolic switch of immune cells upon activation with the ensuing increased lactate production [6]. Similarly, the association of HCAR1 with various cancer types aligns with previous findings [20]. While HCAR1 may not be a determinant factor in these diverse diseases, modulating its signaling could potentially offer a safe adjunct treatment for a wide range of conditions. HCAR1 is thus an attractive therapeutic target with broad applicability.

### Supplementary Information

**Additional file 1: Fig. Supplementary 1.** 3-Chamber social behavior test. a-f) Different parameters of motor behavior do not show any deficiency in locomotion. The quantifications are the cumulative score of each behavior during all 3 phases of the test from all 3 chambers.

**Additional file 2: Fig. Supplementary 2.** Elevated plus maze test. a-f) Different parameters of motor behavior do not show any deficiency in locomotion. The quantifications are the cumulative score of each behavior during the whole test.

**Additional file 3.**

**Additional file 4.**

## Data Availability

The dataset supporting the conclusions of this article is available in GEO repository with accession number of GSE210470.
