# Supplementary figures and images for: Lack of HCAR1, the lactate GPCR, signaling promotes autistic-like behavior
Source: Cell Commun Signal. 2023 Nov 9;21:196. doi: 10.1186/s12964-023-01188-z (PMC10634184; doi:10.1186/s12964-023-01188-z)

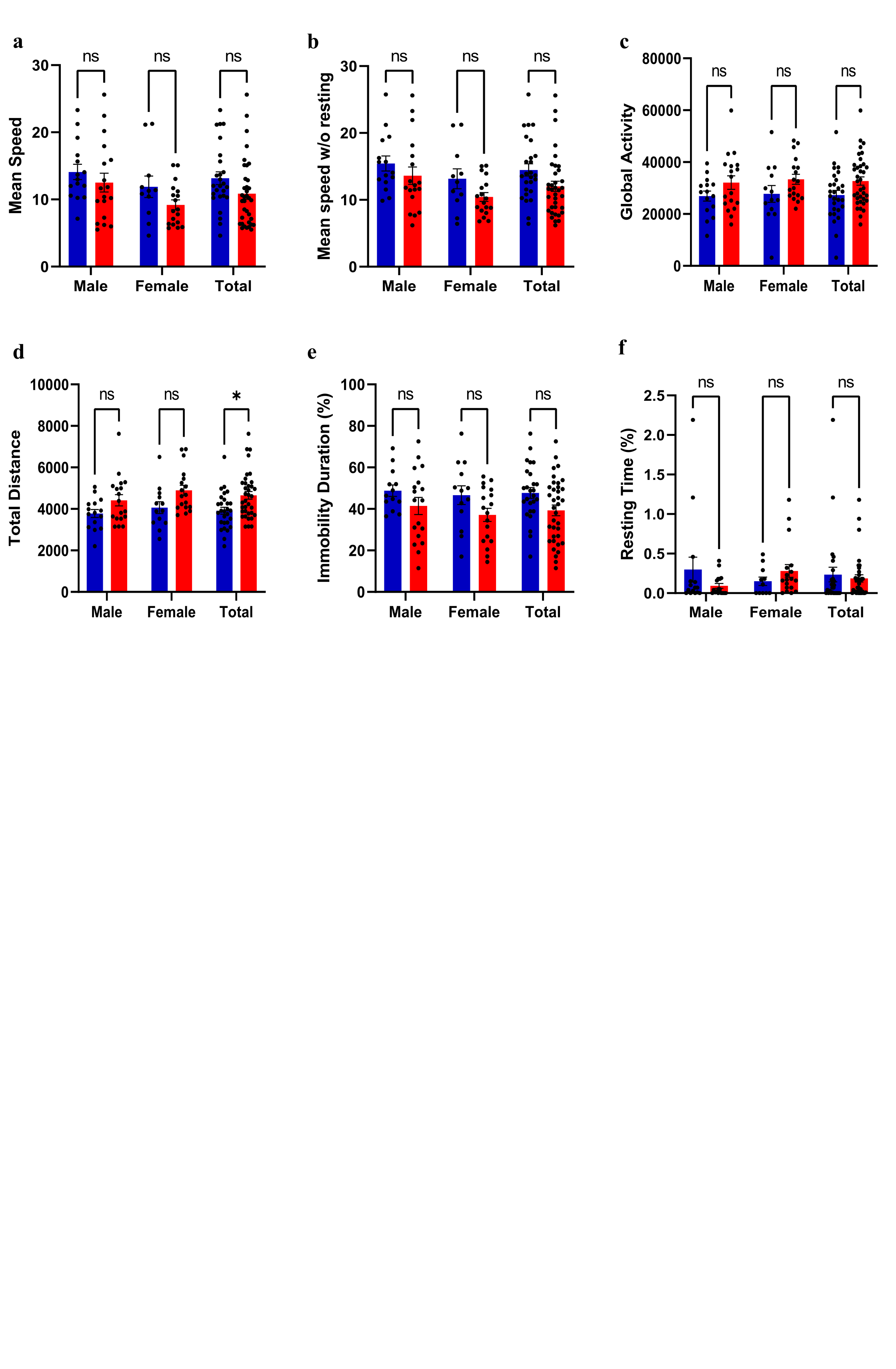

Supplement: Supplementary file 2 — Additional file 1: Fig. Supplementary 1. 3-Chamber social behavior test. a-f) Different parameters of motor behavior do not show any deficiency in locomotion. The quantifications are the cumulative score of each behavior during all 3 phases of the test from all 3 chambers. [file 12964_2023_1188_MOESM1_ESM.tiff]

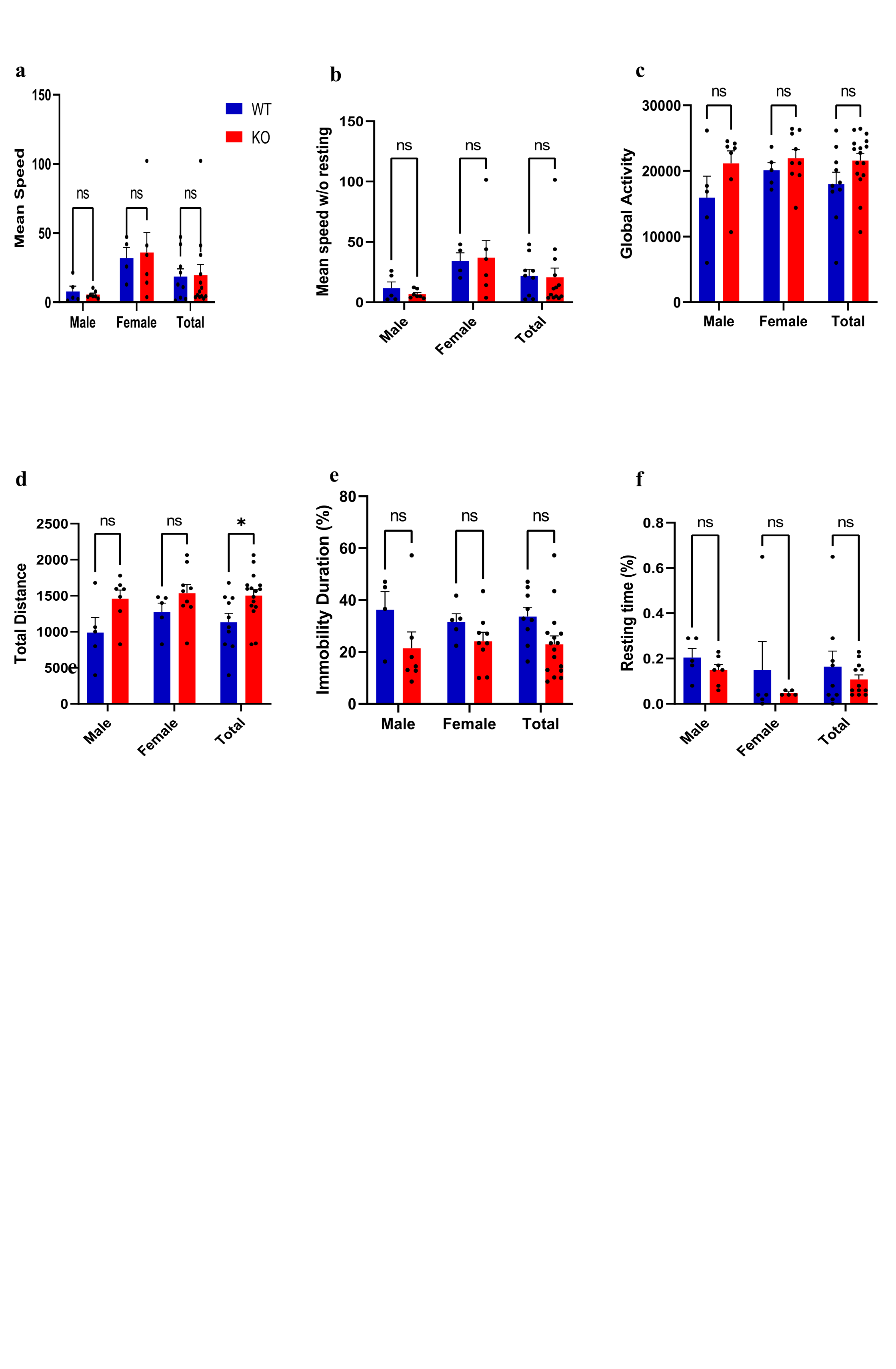

Supplement: Supplementary file 3 — Additional file 2: Fig. Supplementary 2. Elevated plus maze test. a-f) Different parameters of motor behavior do not show any deficiency in locomotion. The quantifications are the cumulative score of each behavior during the whole test. [file 12964_2023_1188_MOESM2_ESM.tiff]
