# Supplementary material for: Lack of HCAR1, the lactate GPCR, signaling promotes autistic-like behavior
Source: Cell Commun Signal. 2023 Nov 9;21:196. doi: 10.1186/s12964-023-01188-z (PMC10634184; doi:10.1186/s12964-023-01188-z)
Supplement: Supplementary file 4 — Additional file 3. [file 12964_2023_1188_MOESM3_ESM.docx]

**Supplemental to:**

**Lack of HCAR1, the lactate GPCR signaling promotes autistic like behavior**

Mohammad Ali Mohammad Nezhady^1,2^, Gael Cagnone^2^, Jean-Sébastien Joyal^2^, Sylvain Chemtob^1,2^

^1^ Program in Molecular Biology, Faculty of Medicine, University of Montreal, Montreal, QC, H3C 3J7, Canada

^2^ Department of Pediatrics, Sainte-Justine University Hospital Research Center, University of Montreal, Montreal, QC, H3T 1C5Canada

**Supplemental Figure legends and Material and Methods:**

**Supplemental Figure legend:**

**Fig. Supplementary 1:** 3-Chamber social behavior test

a-f) Different parameters of motor behavior does not show any deficiency in locomotion. The quantifications are the cumulative score of each behavior during all 3 phases of the test from all 3 chambers.

**Fig. Supplementary 2:** Elevated plus maze test

a-f) Different parameters of motor behavior does not show any deficiency in locomotion. The quantifications are the cumulative score of each behavior during the whole test.

**Material & Method:**

*Transcriptomic:*

Equal number of cells were plated in 10cm Petry dish and were grown to reach a density of 70-80% confluency. A volume lactate for a final concentration of 10mM for experimental groups and equal volume of PBS for control group were added to the cell media and incubated to 6 hours. We extracted RNA with RNeasy mini Kit (Qiagen Cat#74104). Sample analysis and sequencing was performed at IRIC genomic platform. Before sequencing, RNA quantity and integrity was validated with Bioanalyzer. 500 ng of RNA was used for library preparation. RNA quality control was assessed using the Bioanalyzer Nano assay on the 2100 Bioanalyzer system (Agilent technologies) with all samples having a RIN above 9,5. Dyna Beads Oligo(dT) (Thermo Fisher) was used for PolyA selection, RNA Hyperprep kit (Roche) for Library preparation, and Illumina dual-index UMI (IDT) for Ligation. Normalization of all diluted libraries were done by qPCR using the KAPA library quantification kit (KAPA; Cat no. KK4973). Sample libraries were pooled to equimolar concentration. Library preparation and sequencing was made at the Institute for Research in Immunology and Cancer’s Genomics Platform (IRIC).

Nextseq 500 illumina system was used for sequencing with a depth of ~35M per sample with single-end 75 cycles. Sample for each group was sequenced at least in triplicates. Base calls were obtained from the Illumina NextSeq 500 sequencer that runs RTA 2.11.3.0 and raw base calls were converted to FASTQ files using bcl2fastq version 2.20 and allowing 0 mismatches in the multiplexing barcode.

*RNA-seq analysis:*

Next-flow pipeline was used for pre-processing the data^1^ with the salmon pseudo-aligner and the star_salmon aligner (reference genome GRCh38). After regressing for batch effect, we used Seuart^2^ to perform differential gene expression analysis between groups with normalized and scaled gene counts. Genes with more or less than 0.5-fold expression change and p < 0.05 were selected for further analysis. fastGSEA^3^ and enrichR^4^ were used to analyze differentially expressed genes. Final gene list was analyzed by Enrichr for diseases association that are significantly enriched in either of the groups.

*Animal experiments:*

All procedures on animal were approved by institutional ethic committee at CR Sainte-Justine Hospital. One month old male and female C57BL/6J background mice with HCAR1 knock out and wild type genotypes were used in this study. *Hcar1^-/-^* mice were generated in Lexicon Pharmaceuticals (The Hoodlands, TX, USA) by a 4-kb IRES-LacZ-Neo cassette insertion in the transmembrane domain 2 coding sequence of *Hcar1* in C57BL/6J mice. Animals were housed in separately 1 week prior to experiments and maintained on standard feeding protocol with 12 hours light and dark cycles with free access to water and food.

The behavioral tests were monitored and assessed using a camera on top of the test instruments. The animal behavior including their movement, resting time, distance traveled, number of entries to each section and etc were video recoded and analyzed with the SMART video tracking software (v#.0, Harvard Apparatus).

*3-Chamber social test:*

The 3-Chamber social test was performed in 3 phases of 10 minutes each with the subject mouse being able to freely move and explore all three chambers. In the first phase, animals were placed in the empty chambered arena without any object to acclimatize with the environment. Then the subject mouse was removed from the arena between the phases and placed back after the new set-up. In the second phase, an empty wire cage was placed in the either of left or right chamber (alternating for every new subject mouse) and another wire cage with an unfamiliar mouse was placed in the opposing chamber. The sociability of mice was assessed in the 2^nd^ phase of this test. In the third phase, a new unfamiliar mouse was placed in the empty cage from the 2^nd^ phase before bringing the subject mouse back. At this phase, the social novelty behavior of animal was assessed. The sociability and social novelty parameters (1^st^ latency entrance, number of entries, resting time, sociability, and social novelty) were measured by the presence of mouse in the interaction zone only (the circled area surrounding the wire cages, depicted in the Fig. 1d). The subject mice were always put in the middle chamber in all phases. Both caged mice were wild type background with same age and sex as the subject mouse but from different home cages and had no prior contact with each other nor the subject mice. The sociability and social novelty behavior were evaluated by quantifying the time that subject mouse spent with the object or each caged mice in their surrounding designated area^5,6^.

*Elevated plus maze:*

We performed elevated plus maze to measure the anxiety-like behavior of mice^7^. Mice were placed in the center of the maze with two opposing open arms and two perpendicular opposing closed arms. Their movement was monitored for 5 minutes by video recording.

*Statistics:*

Statistical analyses were performed using Prism 9.0 (GraphPad Software). Differences between groups were assessed with Analysis of variance (ANOVA) followed by Bonferroni post hoc correction test with * *P* < 0.05, ** *P* < 0.01, ****P* < 0.0001 significance levels.

**References:**

1. Ewels, P. A. *et al.* The nf-core framework for community-curated bioinformatics pipelines. *Nat. Biotechnol.* **38**, 276–278 (2020).

2. Hao, Y. *et al.* Integrated analysis of multimodal single-cell data. *Cell* **184**, 3573-3587.e29 (2021).

3. Korotkevich, G. *et al.* Fast gene set enrichment analysis. *bioRxiv* 60012 (2021) doi:10.1101/060012.

4. Xie, Z. *et al.* Gene Set Knowledge Discovery with Enrichr. *Curr. Protoc.* **1**, (2021).

5. Bey, A. L. & Jiang, Y. Overview of Mouse Models of Autism Spectrum Disorders. *Curr. Protoc. Pharmacol.* **66**, (2014).

6. Arakawa, H. From Multisensory Assessment to Functional Interpretation of Social Behavioral Phenotype in Transgenic Mouse Models for Autism Spectrum Disorders. *Front. Psychiatry* **11**, (2020).

7. Silverman, J. L., Yang, M., Lord, C. & Crawley, J. N. Behavioural phenotyping assays for mouse models of autism. *Nat. Rev. Neurosci.* **11**, 490–502 (2010).
